# A Novel Comprehensive Clinical Stratification Model to Refine Prognosis of Glioblastoma Patients Undergoing Surgical Resection

**DOI:** 10.3390/cancers12020386

**Published:** 2020-02-07

**Authors:** Tamara Ius, Fabrizio Pignotti, Giuseppe Maria Della Pepa, Giuseppe La Rocca, Teresa Somma, Miriam Isola, Claudio Battistella, Simona Gaudino, Maurizio Polano, Michele Dal Bo, Daniele Bagatto, Enrico Pegolo, Silvia Chiesa, Mauro Arcicasa, Alessandro Olivi, Miran Skrap, Giovanni Sabatino

**Affiliations:** 1 Neurosurgery Unit, Department of Neuroscience, Santa Maria della Misericordia University Hospital, 33100 Udine, Italy; skrap@asuiud.sanita.fvg.it; 2 Department of Neurosurgery, Mater Olbia Hospital, 07026 Olbia, Italy; fabrizio.pignotti@materolbia.com (F.P.); giovanni.sabatino@policlinicogemelli.it (G.S.); Giuseppe.larocca@policlinicogemelli.it (G.L.R.); 3 Institute of Neurosurgery, Catholic University, 00168 Rome, Italy; giuseppemaria.dellapepa@policlinicogemelli.it (G.M.D.P.); alessandro.olivi@policlinicogemelli.it (A.O.); 4 Division of Neurosurgery, Department of Neurosciences, Reproductive and Odontostomatological Sciences, Università degli Studi di Napoli Federico II, 80131 Naples, Italy; teresa.somma85@gmail.com; 5 Department of Medicine, Santa Maria della Misericordia University Hospital, 33100 Udine, Italy; miriam.isola@uniud.it (M.I.); claudio.battistella@uniud.it (C.B.); 6 Institute of radiology, Fondazione Policlinico Universitario A. Gemelli IRCCS, 00168 Rome, Italy; simona.gaudino@policlinicogemelli.it; 7 Experimental and Clinical Pharmacology Unit, Centro di Riferimento Oncologico di Aviano (CRO) IRCCS, 33081 Aviano, Italy; mpolano@cro.it (M.P.); mdalbo@cro.it (M.D.B.); 8 Neuroradiology Unit, Department of Diagnostic Imaging ASUIUD Udine, 33100 Udine, Italy; daniele.bagatto@asuiud.sanita.fvg.it; 9 Institute of Pathology, Santa Maria della Misericordia University Hospital, 33100 Udine, Italy; enrico.pegolo@asuiud.sanita.fvg.it; 10 Radiation Oncology Unit, Fondazione Policlinico Universitario A. Gemelli IRCCS, 00168 Rome, Italy; Silvia.chiesa@policlinicogemelli.it; 11 Department of Oncology, Centro di Riferimento Oncologico di Aviano (CRO) IRCCS, 33081 Aviano, Italy; marcicasa@cro.it

**Keywords:** glioblastoma prognosis, overall survival, extent of resection, random forest, Decision tree, personalized precision oncology

## Abstract

Despite recent discoveries in genetics and molecular fields, glioblastoma (GBM) prognosis still remains unfavorable with less than 10% of patients alive 5 years after diagnosis. Numerous studies have focused on the research of biological biomarkers to stratify GBM patients. We addressed this issue in our study by using clinical/molecular and image data, which is generally available to Neurosurgical Departments in order to create a prognostic score that can be useful to stratify GBM patients undergoing surgical resection. By using the random forest approach [CART analysis (classification and regression tree)] on Survival time data of 465 cases, we developed a new prediction score resulting in 10 groups based on extent of resection (EOR), age, tumor volumetric features, intraoperative protocols and tumor molecular classes. The resulting tree was trimmed according to similarities in the relative hazard ratios amongst groups, giving rise to a 5-group classification tree. These 5 groups were different in terms of overall survival (OS) (*p* < 0.000). The score performance in predicting death was defined by a Harrell’s c-index of 0.79 (95% confidence interval [0.76–0.81])**.** The proposed score could be useful in a clinical setting to refine the prognosis of GBM patients after surgery and prior to postoperative treatment.

## 1. Introduction 

Glioblastoma (GBM) is the most common primary malignant central nervous system (CNS) tumor in adults, representing about 25% of primary CNS tumors and 50%–55% of adult gliomas [1,2,3]. The current standard of care for GBM includes maximal safe surgical resection followed by concomitant chemoradiation therapy and adjunct chemotherapy [4,5,6,7,8]. Despite decades of advances in surgery and discovery in the molecular landscape, encouraging outcomes are not typically observed; patients diagnosed with these tumors generally have a dismal prognosis and poor quality of life as the disease progresses. The median survival time has been reported to be less than 15 months in cases. Survival longer than 3 years and 5 years have been reported for approximately 3%–5% and 0.5% of GBM patients, respectively. There is thus a pressing need to identify new systemic therapies [9,10,11]. The variety in overall survival and response to treatment in GBM is largely due to the high heterogeneity of GBM with a different distribution of aggressive biological traits across tumors, as well as within a single tumor [12,13,14]. To classify GBM cases according to this heterogeneity, different prognostic factors have been suggested for GBM, including age, performance status, specific molecular markers [e.g., MGMT methylation (O^6^-methylguanine-DNA methyl-transferase), mutation of IDH1, IDH2(isocitrate dehydrogenase) or TERT (telomerase reverse transcriptase), 1p19q codeletion, overexpression of EGFR (epidermal growth factor receptor)], the size of necrosis and the extent of resection (EOR) [15,16,17,18,19,20,21,22]. The role of EOR in improving survival in patients with GBM has widely been demonstrated, with more extensive resections providing added advantages [8,9,16,18,19,23,24,25,26,27,28,29,30,31,32,33].

In this context, survival benefit based on extent of tumor resection has been reported to be as low as 78% and the greatest survival advantage has been seen in patients with EOR >95% [9]. Despite the infiltrative nature of this tumor, it still remains unclear if the resection beyond the contrast enhancement portion of the tumor translates into improved outcomes for patients with GBM [23].

In a clinical setting, the need for classification tools based on the prognostic stratification of GBM cases undergoing surgical protocols is of increasing importance. Numerous attempts have been developed to classify GBM patients, which include combination models of clinical, molecular and radiomic variables used in daily clinical practice [34,35,36,37,38].

Given the importance of each individual factor, it is often difficult to establish how these interact with each other and how they impact survival in the complexity of the clinical settings. In other words, classical survival models do not concomitantly evaluate multiple variables and establish the burden of different combinations of determinants on survival.

In the present investigation, we proposed a novel prognostic model comprehensively evaluating clinical, surgical volumetric and molecular factors to define prognosis of GBM-affected patients undergoing surgery.

## 2. Results

Demographic, clinical, neurophysiological and radiological features of the study population are summarized in Table A1 and Table A2.

### 2.1. Survival Analysis and Risk Factors

**Figure 1:**
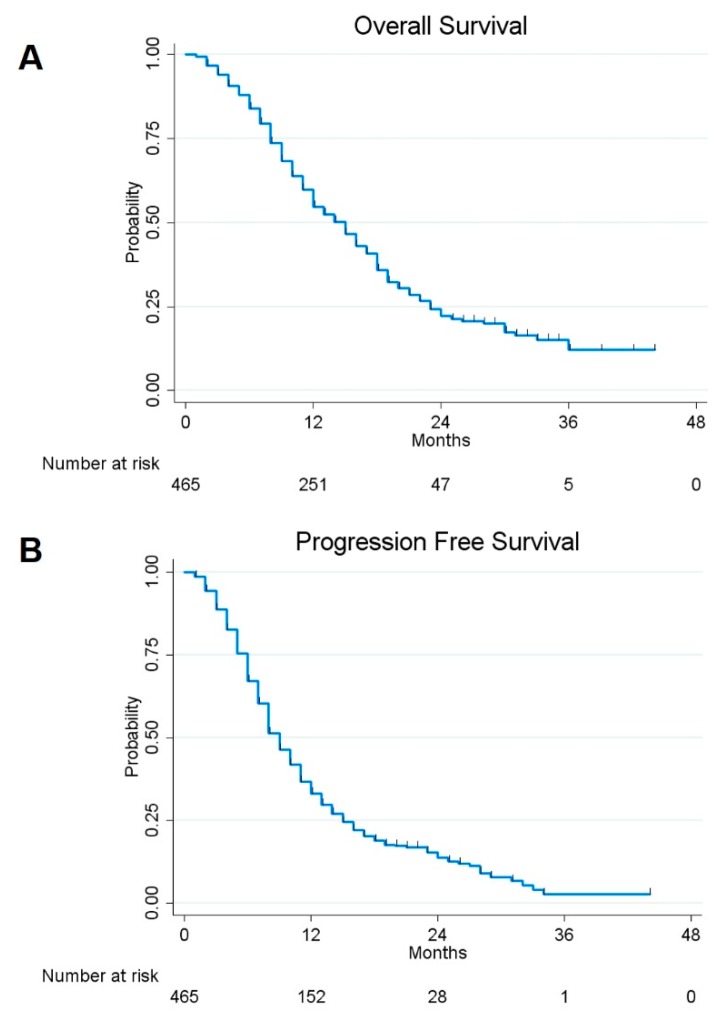
Kaplan–Meier curves displaying overall survival (OS) (**A**) and progression-free survival (PFS) (**B**) in the whole sample of 465 glioblastoma (GBM) included in the study.

The 1- and 2- year overall survival (OS) and progression-free survival (PFS) rates for the assessed patients were estimated to be 54.78% and 22.28%, and 33.05% and 13.82%, respectively (Figure 1).

**Figure 2:**
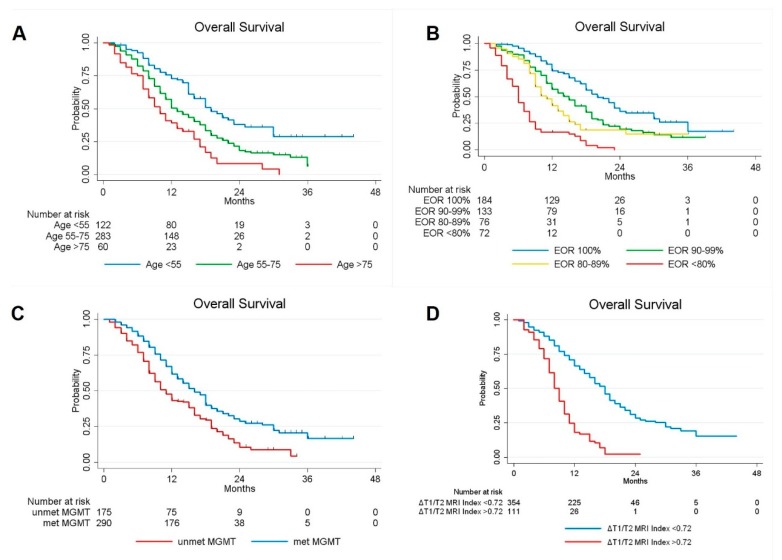
Kaplan-Meier curves displaying OS of GBM patients according to Age (**A**); EOR (**B**); MGMT promoter methylation status(**C**); and preoperative ΔT1/T2 MRI Index (**D**).

Univariate analysis showed a significant better survival in patients with a younger age (*p* = 0.000), higher EOR (*p* = 0.000), methylated MGMT promoter (*p* = 0.000), mutation of IDH1/IDH2 genes (*p* = 0.033), presence of lower residual tumor (*p* = 0.000) and lower preoperative ΔT1/T2 MRI Index *(p =* 0.000) (Figure 2). Gender, tumoral side and tumoral site, however, did not statistically influence OS.

At multivariate Cox analysis, considering the variables with a significant p value in univariate analysis, EOR (*p* = 0.000), age (*p* = 0.000), MGMT methylation status (*p* = 0.000) and preoperative ΔT1/T2 MRI Index (*p* = 0.000) were confirmed as independent predictors for OS (Table 1).

**Figure 3:**
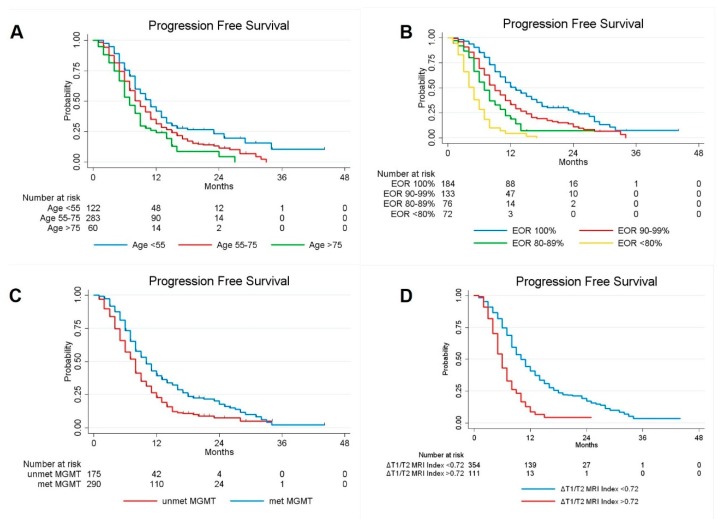
Kaplan-Meier curves displaying PFS of GBM patients according to Age (**A**); EOR (**B**): MGMT promoter methylation status (**C**); and preoperative ΔT1/T2 MRI Index (**D**).

Similarly, when PFS was considered, univariate Cox regression analyses confirmed age (*p* = 0.000), EOR (*p* = 0.000), methylation status of MGMT promoter (*p* = 0.000) and preoperative ΔT1/T2 MRI Index (*p* = 0.000) as factors influencing the tumor progression. By performing multivariate Cox analysis considering the variables with a significant p value in univariate analysis, EOR (*p* = 0.000), age (*p* = 0.002), methylation status of MGMT promoter (*p* = 0.000) and preoperative ΔT1/T2 MRI Index (*p* = 0.000) were confirmed as independent predictors for PFS, however, no correlation was observed with other observed variables such as sex, tumor size and site, IDH-1 status and Ki67% (Figure 3, Table 2).

### 2.2. Classification and Regression Tree (CART) Model

**Figure 4:**
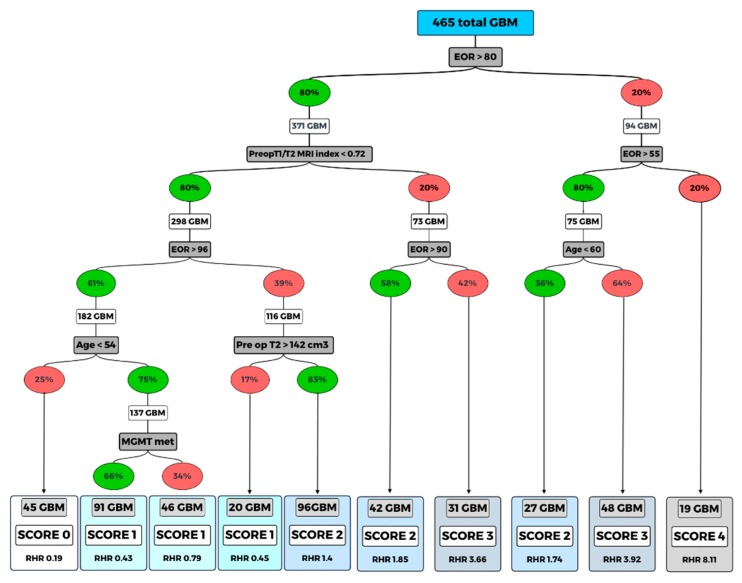
Random forest (classification and regression tree, CART).

In order to create a prognostic model comprehensively evaluating clinical, molecular and treatment-associated factors to stratify GBM-affected patients undergoing surgery, we used a classification and regression tree (CART) approach. The algorithm relied on the clinical variables that showed a significant impact as independent predictor factors in multivariate analysis (age, EOR, MGMT methylation status, preoperative ΔT1/T2 MRI Index, preoperative volumetric tumor volume on T2- weighted images. (Figure 4). The application of the CART analysis led to the definition of 10 terminal nodes (Figure 4). According to the relative hazard ratio (RHR) obtained by performing the CART analysis, a clinical predictive score (GAPS = GBM-associated prognostic score) was elaborated. In detail, a score from 0 to 4 was then assigned to the 11 terminal nodes (score 0, assigned to the nodes with RHR ≤ 0.40; score 1 assigned to the nodes with RHR between 0.40 and 1.00; score 2 assigned to the nodes with RHR between 1.00 and 2.00; score 3 assigned to the nodes with RHR between 2.00–4.00; score 4 was assigned to the nodes with RHR > 4.00). Each score group was defined based on the following characteristics: Score 0: patients with EOR > 96%, preoperative ΔT1/T2 MRI Index < 0.72 and age < 53; Score 1: patients with EOR > 96%, T1/T2 < 0.72 and age > 53; patients with EOR between 81% and 95% if they have a preoperative ΔT1/T2 MRI Index < 0.72 and preoperative T2-weight volume > 147 cm3; Score 2: patients with EOR between 81% and 95%, preoperative ΔT1/T2 MRI Index < 0.72 and preoperative T2-weight volume < 147 cm3; patients with EOR > 80%, preoperative ΔT1/T2 MRI Index > 0.72, if they have EOR between 91% and 100%; patients with EOR between 56% and 80% if they are aged < 59; Score 3: patients with preoperative ΔT1/T2 MRI Index > 0.72 and EOR between 81% and 90%; patients with EOR between 56% and 80% if they have age > 60; Score 4: all patients with EOR < 55%. The obtained 5 groups of GBM cases were associated with different OS: score 0 group included 45 cases (accounting to the 9.68% of cases), score 1 included 157 cases (33.76%), score 2 included 165 cases (35.48%), score 3 included 79 cases (16.99%), and score 4 included 19 cases (4.09%). 

Once the scores were obtained, a univariate Cox regression was performed to evaluate the predictive ability of the score.

Compared to score 0 (low risk), score 1 had hazard ratio (HR) = 2.6 (95% CI: [1.4–5.0], *p* = 0.003); score 2 had HR = 9.6 (95% CI: [5.1–18.3], *p* = 0.000); score 3 had HR = 28.1 (95% CI: [14.4–54.7], *p* = 0.000); score 4 had HR = 85.4 (95% CI: [38.5–189.3], *p* = 0.000).

The goodness of fit of the score model in predicting death was estimated with a Harrell’s c-index of 0.78 (95% IC [0.76–0.81]). The 1-yr estimated OS was computed for each score category (Table 3).

The algorithm relied on six clinical variables that shows the interaction between the significant variables at multivariate analysis (age, EOR, MGMT methylation status, preoperative ΔT1/T2 MRI index, pre-operative volumetric tumor volume on T2-weighted images and intraoperative protocol).

The score (GAPS) from 0 to 4 was then assigned to the 10 terminal nodes thus defined based on the relative hazard ratio (RHR). Percent values indicated in the in green ovals represent the presence of the variable considered; the red color indicates absence of that variable.

### 2.3. Treatment at Tumor Progression

In this study, a population of 369 cases experienced tumor progression; 298 were treated with salvage treatments, while the others with supportive care (SC).

Among patients treated with salvage treatments (298), the impact of treatment type (TMZ, second surgery, TMZ + RT, RT alone, photemustine-lomustine) on OS was analyzed.

At tumor progression, TMZ was administered in 215 patients, 43 patients underwent a second surgery. Twenty-four patients were treated with TMZ + RT, 8 patients with RT alone and 8 patients with photemustine-lomustine.

By applying the Kaplan–Meier survival estimates and the logrank test, there were no differences in survival on the basis of the treatment adopted at tumor progression (*p* = 0.236 considering all the subgroups taken separately; *p* = 0.199 combining TMZ + RT, RT and photemustine-lomustine). The Cox regression analysis also confirmed this evidence taking the TMZ treatment as a reference and combining the others salvage treatments. There was no association for intervention (HR = 0.760 [95% CI: 0.513–1.126], *p* = 0.172); TMZ+RT (HR = 0.670 [95% CI: 0.394–1.140], *p* = 0.140); RT (HR = 1.280 [95% CI: 0.628–2.608], *p* = 0.496); photemustine-lomustine (HR = 0.643 [95% CI: 0.263–1.571], *p* = 0.332).

One-year estimated PFS was computed in all generated GAPS class scores, resulting in being significantly different in each score class (Kruskall–Wallis test, *p* = 0.001) (Table 4).

## 3. Discussion

Despite decades of therapeutic, surgical and genetics refinements, GBM still remains the highest-grade malignant primary tumor of the central nervous system with an extremely poor prognosis [4,10,11,19,39].

Age, performance status, extent of surgical resection and MGMT methylation status are well known prognostic factors for GBM patients [4,21,22,23,24,25,26,27,28,29,30,31,32,33,34,39,40,41,42,43,44]. Nevertheless, the high degree of clinical/molecular heterogeneity found among GBM patients do not generally allow us to correctly classify GBM patients with the use of a single predictor or a few predictors. There is thus an increasing need of comprehensive predictive classification models, which concomitantly evaluate multiple clinical/molecular/radiomic biomarkers. Moreover, given the high degree of heterogeneity in survival rates among GBM patients, it becomes essential to use tools that are capable of considering the possible interaction between the significant independent survival variables as further possible source of differences in the survival outcome. 

In the present study, we set up a prognostic model that comprehensively evaluated clinical, molecular and treatment-associated factors to stratify GBM-affected patients undergoing surgery using a random forest approach (CART). This model generated an integrative visualization of risk factors, giving rise to an easy and immediate interactive interpretation of results. 

The analysis consisted of 3 main steps: first, the most informative variables were identified; then, a decision tree algorithm was applied to differentiate the survival and lastly the GAPS score was generated. 

Five variables were selected as the most informative amongst the 20 variables considered. The highest classification accuracy included age, preoperative tumor volume computed on T2-wheighted MRI, preoperative ΔT1/T2 MRI Index, EOR, and MGMT methylation status. The interactions were analyzed using the CART model. 

There has been an increasing number of volumetric investigations highlighting the association between the EOR and survival [8,9,16,18,19,23,25,33]. Nowadays an increasing variety of neurosurgical methods are available (e.g., frameless navigational systems, intraoperative imaging, ultrasonography, and functional mapping) to achieve the optimum balance between a maximal resection and a safe resection.

By performing the random forest approach, the EOR was placed on top of the decision tree. Specifically, the obtained results showed that cases with EOR >80% were associated with a longer survival rate. This finding is in keeping with previously published retrospective investigations suggesting that at least 70%–78% of the contrast-enhancing tumor volume represents the ideal resection target for survival benefit [9,25]. Sanai and colleagues were the first that have highlighted the importance of EOR threshold in GBM survival [9]. In line with their contribution, our study reported the best survival rate in patients with an EOR higher than 96% with estimated 1-yr OS of 92%.

Another important finding highlighted by the CART was the relevant impact on GBM prognosis for preoperative volumetric radiological features. The hallmarks of GBM on MRI are the contrast-enhancing tumor with its central necrosis and surrounding peritumoral edema. Each tumoral component could represent a potential imaging marker to predict the OS. 

There is still open discussion among neuroncologists regarding which tumoral component has to be considered (e.g., contrast enhancement, peritumoral edema, central necrosis [13,23,45]. 

In this investigation, the preoperative MRI index based on the ΔT1/T2 MRI ratio was computed as previously described [45], resulting as being an independent predictor both for OS and PFS. Specifically, patients with a preoperative ΔT1/T2 MRI Index ratio close to 1 had a poor prognosis compared to those with preoperative T1/T2 MRI ratio close to 0, in other words lesions with ratio close to 1 should have more aggressive growth, as opposed to lesions with a ratio close to 0. However, we cannot ultimately identify which aspects of tumoral behavior determine the preoperative ΔT1/T2 MRI Index and given the wide heterogeneity of GBM, future investigations based on texture features from multiparametric MRI and next generation sequences analysis, may further clarify this issue.

Elderly age negatively affected the prognosis only in cases of limited resection, supporting the role of surgery in fit older patients when a safe and large resection can be planned [42,43,44].

Regarding the molecular features, the MGMT methylation status positively influenced the prognosis only in patients younger than 54 years with an EOR higher than 96%, thus suggesting the possibility of other genetic abnormalities potentially affecting survivals of GBM patients.

CART analysis provided 10 terminal nodes; the RHR of which were used to generate the score (GAPS) with the purpose of facilitating the survival stratification before patients were discharged postoperatively.

GAPS were elaborated to detect the impact of variables interaction on the overall survival giving rise to an easy and immediate interpretation score. Patients belonging to score 0 (preoperative ΔT1/T2 MRI Index < 0.72; EOR > 96%; Age < 53) had the better survival with a 3 years estimated OS of 25%, otherwise, the worse survival was for patients with score 3 and 4 (preoperative ΔT1/T2 MRI Index > 0.72; EOR 81%–90%, or EOR: 56%–80%; age > 60; or EOR < 55) with 1 year-estimated OS of 11.58% and 5.26% respectively after surgery being equal with regards to the post-operative treatments.

The novelty of this approach is that the focus is on the interaction of different factors rather than the single determinant. This allows the building of a model as close as possible to the real clinical setting.

The GAPS score could be useful in a day-to-day clinical environment and in a research setting to draw future prospective clinical trials. Moreover, GAPS score could be useful when deciding and discussing prognosis to better handle the entire GBM management. 

We aware that our study has several limits, which include the retrospective nature of the investigation and the different treatments performed at tumor progression. Moreover, the retrospective study did not permit a standardized follow-up.

The GAPS score could be useful in a day-to-day clinical environment and in a research setting to draw future prospective clinical trials. Moreover, GAPS score could be adopted in discussing prognosis to better handle the entire GBM management. 

We aware that our study has several limits, which include the retrospective nature of the investigation and the different treatments performed at tumor progression. Moreover, the retrospective study did not permit a standardized follow-up.

The statistical limitations of such a retrospective analysis are well known and cannot be completely controlled with any statistical model.

Treatments at GBM relapse represents a crucial issue. The recurrence of GBM is inevitable, in which management often tend to be unclear and case-dependent. Although re-radiation, re-resection, bevacizumab, and chemotherapy are still the most widely used therapies for treating recurrent GBM, the clinical benefit from these treatments is still not well established [46,47,48,49,50].

It is well known that to improve the prediction models, salvage treatments information should be updated in the analysis at the time of tumor progression.

Longer PFS resulted in late tumor recurrence and consequently in better OS [46]. 

In this investigation, patients with lower GAPS score had a longer PFS and consequent better OS. The predictive survival score computed in this investigation can, thus, be considered as an indirect measure of tumor progression. Patients with GAPS score of 0 had a better survival and prolonged PFS determined by the combination of radiological, surgical and molecular factors before tumor recurrence. The score was analyzed based on the characteristics of the patients included in the model. This tool provides information regarding a more or less rapid risk of progression before the administration of salvage treatments at tumor progression itself. For this reason, the salvage treatments cannot be considered. In addition, progression time is different in the different GAPS score classes and time dependent analysis should be applied to evaluate the effect of salvage treatments on OS. 

Data regarding selection criteria adopted at tumor recurrence to plan the salvage treatment were not available. Each patient underwent an individualized management at tumor progression. We have not developed standardized protocols for treatments at tumor progression, which is another drawback of this study that requires future investigation.

Future prospective multicenter studies in a larger group of patients with a long follow-up are needed to overcome the inherent limitations of a retrospective study and to confirm the potential clinical usefulness of this tool in the management of GBM patients. Genetic studies could be integrated in this preliminary model in order to improve the accuracy of the score in the stratification of GBM patient prognosis.

## 4. Materials and Methods 

A shared co-operative record databased 520 adult patients who underwent surgery for newly diagnosed GMB between January 2015 and December 2018; 465 GBM patients were enrolled in the case cohort according to the following inclusion criteria: age ≥ 18 years; no previous surgery; no preoperative chemo- or radiotherapy; objective evaluation of preoperative tumor volume on MRI images in DICOM format based on post-contrast T1-weighted MRI sequences and T2-weighted MRI sequences; objective estimation of EOR on post-contrast T1-weighted MRI sequences; revision of histopathological specimens by using the new 2016 World Health Organization (WHO) Classification of Tumors of the Central Nervous System [51]; MGMT promoter methylation and IDH1/IDH2 mutation status assessment. Cases were excluded from the case cohort if one or more of the following criteria were present: incomplete imaging data, follow-up interval, and multicentric tumors. Clinical, histopathological and molecular data were collected at the time of diagnosis from medical records. No central histopathological review and no additional molecular analyses were performed for the purpose of the study

Histological examination, immunohistochemistry for Ki67 and IDH1R132H, analysis of the genetic status of O6-methylguanine-DNA-methyltransferase (MGMT) promoter and isocitrate dehydrogenase (IDH1/2) genes were performed as previously described. Gliomas were defined as methylated when the average percentage of methylation of CpG islands was ≥8% [52].

Patients were clinically evaluated both prior to discharge, and at subsequent 4-monthly intervals. Patients that exhibited no clinical improvement by 6 months after surgery were considered to have a permanent deficit. In the follow-up period MRI images were obtained at regular (4-monthly) intervals.

The present study was approved by the local Ethics Committee (protocol N. 0036566 /P/ GEN/ EGAS, ID study 2538). Written informed consent was obtained for surgery. Considering that the study was retrospective, written consent to participate in the study was not applicable.

### 4.1. Volumetric Analysis 

All pre and postoperative tumor segmentations were performed manually across all MRI slices using the OsiriX software tool [53].

The achieved EOR in each case was objectively evaluated using preoperative and postoperative MRI images (DICOM format), based on the contrast area of post-contrast T1 MRI sequences, using the below formula: (Pre-operative tumor volume – Post-operative tumor volume)/Pre-operative tumor volume) [54].

With the aim of evaluating the role of tumor growing pattern on OS, a novel predictive preoperative MRI index was defined as follows T1/T2 = preoperative volumetric tumor volume on post contrast T1-weighted images/ preoperative volumetric tumor volume on T2-weighted images.

### 4.2. Post-Operative Treatment

After surgery, all patients were treated with combinations of concomitant adjuvant radiotherapy and chemotherapy, followed by adjuvant chemotherapy, as recommended by Stupp [5]. 

External-beam (either conformal or stereotactic) radiotherapy was used to administer a total dose of 60 Gy (delivered in 30 fractions of 2 Gy over a 6-week period), followed by adjuvant oral chemotherapy with temozolomide (75 mg/m^2^/day, 7 days/wk). Four weeks after the end of this treatment protocol, patients underwent at least six cycles of consolidation chemotherapy with oral temozolomide (150–200 mg/m^2^/day, for 5 days/28 days).

### 4.3. Statistical Analysis

Categorical variables were reported as percentages, continuous variables were reported as mean ± standard deviation or median and range as appropriate, according with the data distribution. Normality of the continuous variables was tested using the Shapiro–Wilk test. The OS time was defined as extending from surgery until patient death; PFS time was defined as extending from surgery until the demonstration of gadolinium enhancement on follow-up imaging. OS and PFS were estimated using the Kaplan–Meier approach. The association between variables and survival distribution was tested using univariate and multivariate Cox proportional hazard models (after verification of proportional hazard assumptions). Patients with unknown survival were censored as of their last scan date. The variables we considered for univariate analysis were age, sex, KPS score, preoperative tumor volume computed on post contrast T1-wheighted images and on T2- weighted images MRI, tumor location, tumor side, EOR, postoperative adjuvant protocol used, IDH ½ mutation, MGMT mutilation status and Ki-67. The EOR was modeled both as a continuous and an ordinal variable (≤79%, 80%–89%, 90%–99%, 100%) in univariate analysis to ensure consistency with the previous 46 studies that focused on the impact of glioma resection in terms of volumes. The preoperative ΔT1/T2 MRI Index was calculated by the ratio between pre- operative tumor volume calculated on post-contrast T1-wheighted images MRI and the pre-operative tumor volume calculated on T2-weighted images MRI. In the univariate Cox regression, the preoperative ΔT1/T2 MRI Index was initially analyzed as a continuous variable. To better understand the variable’s association pattern, the Cox regression was then applied to the quintiles splitted variable. Subsequently, the variable was dichotomized using a cut-off we identified at the quintile that showed a significant hazard ratio. The variables resulted in being significantly associated in the univariate model with *p* < 0.05. All statistical analyses were performed by Stata/IC 13.0 (StataCorp LP, College Station, TX, USA).

### 4.4. Classification and Regression Tree (CART) Method 

To determine subgroups patients with different clinical prognosis, we used the decision tree model using the CART method [55,56].

This method is a machine learning model, composed of hierarchic decision rules involving optimal cutoff values that recursively split independent factors into different groups. The groups of individuals are called nodes, and form a branch node tree. Terminal nodes are groups of individuals that cannot be further subdivided on the basis of the established parameters (minimum size of subgroup, minimum number of events, maximum p-value required) to proceed in further subdivisions. The CART algorithm was performed on the entire sample (465 cases). In our study, nodes were required to have a minimum size of 15 patients, a minimum of 10 events and a maximum *p*-value of 0.05. Factors initially introduced into this CART analysis are the following: EOR, preoperative ΔT1/T2 MRI Index, age, MGMT methylation, pre-operative volumetric tumor volume on T2-weighted images and intraoperative protocol. Once the regression tree was generated, the nodes of the terminal branches were pruned (aggregated) on the basis of their relative hazard ratios (RHRs) in order to obtain final groups with homogeneous mortality risk. The final groups were converted in a score ordered according to their hazard ratios (HRs).

Differences in terms of overall survival probability among the score categories were investigated using univariate Cox regression analysis. The performance of the score in predicting time to death was estimated through Harrell’s c-index [57]. All statistical analyses were performed by Stata/IC 13.0 (StataCorp LP, College Station, TX, USA).

## 5. Conclusions

Nowadays, the current standard of care for GBM still includes maximal safe surgical resection followed by concomitant chemoradiation therapy and adjunct of chemotherapy. The high degree of clinical heterogeneity found among GBM highlights a rising need for comprehensive predictive classification models concomitantly evaluating multiple clinical/molecular/radiomic biomarkers. The CART prediction model allowed to elaborate a novel comprehensive clinical score (GAPS) to stratify prognosis of glioblastoma patients undergoing surgical resection. Although GAPS needs to be validated in further multicenter studies, it could facilitate the survival-risk grading, guiding clinicians in the decision-making process. 

## Figures and Tables

**Table 1:** Univariate and multivariate analysis of OS in GBM patients.

Variable	Univariate Analysis			Multivariate Analysis		
	Hazard Ratio	95% CI	*p*-Value	Hazard Ratio	95% CI	*p*-Value
Age (yrs)	1.029	1.018–1.040	**0.000**	1.028	1.017–1.039	0.000
Sex						
Male	1					
Female	0.900	0.713–1.137	0.377			
Side						
Left	1					
Right	1.124	0.898–1.406	0.308			
Tumor Site						
Precentral	1					
Retrocentral	1.092	0.825–1.446	0.539	0.954	0.718–1.267	0.745
Temporal + Insular	1.250	0.961–1.626	0.097	1.286	0.986–1.677	0.063
Radiological Features						
Ependymal involvement (*yes vs no*)	1.135	0.890–1.448	0.309			
Corpus Callosum involvement (*yes vs no*)	1.012	0.799–1.281	0.922			
Necrotic-cystic component (*yes vs no*)	0.923	0.725–1.176	0.517			
Midline shift (*yes vs no*)	0.970	0.775–1.214	0.789			
Preoperative Tumoral Volume computed on postcontrast T1-weighted images, cm^3^	1.001	0.996–1.006	0.652			
Preoperative Tumoral Volume computed on T2-weighted images, cm^3^	0.993	0.991–0.995	0.000	0.997	0.995–1.000	0.058
Preoperative ΔT1/T2 MRI Index	1.022	1.017–1.026	0.000	1.016	1.009–1.022	0.000
Residual tumor, cm^3^	1.085	1.067–1.103	0.000	0.962	0.925–1.000	0.053
EOR (continuous variable)	0.946	0.938–0.954	0.000	0.937	0.923–0.950	0.000
EOR (categorical variable)						
EOR = 100%	1					
99% ≤ EOR ≤ 90%	1.755	1.314–2.343	0.000			
89% ≤ EOR ≤ 80%	2.477	1.757–3.492	0.000			
EOR ≤ 79%	6.300	4.537–8.748	0.000			
Biological Features						
MGMT promoter methylation (*yes vs no*)	0.605	0.482–0.760	0.000	0.606	0.480–0.765	0.000
IDH 1/2 mutation (*yes vs no*)	0.638	0.423–0.964	0.033	0.925	0.605–1.416	0.721
Ki67	1.001	0.995–1.007	0.725			

Table showing the influence of different factors on the OS rates as per univariate survival analysis and multivariate analysis on the entire GBM patients cohort. (*p*-value < 0.05 at Log-rank test). Boldfacing values represent statistical significant results (*p* < 0.05). CI = confidence interval; *p*-value = level of marginal significance; MRI = magnetic resonance image; preoperative ΔT1/T2 MRI Index = ratio between pre-operative tumoral volume on post-contrast T1-weighted and T2 weighted images; EOR = extent of resection; CWs = Carmustine Wafers; RT = radiotherapy; CT = chemotherapy; MGMT = O^6^-methylguanine-DNA methyl-transferase; IDH = isocitrate dehydrogenase; OS = overall survival.

**Table 2:** Univariate and Multivariate Analysis of PFS in GBM patients.

Variable	Univariate Analysis			Multivariate Analysis		
	Hazard Ratio	95% CI	*p*-Value	Hazard Ratio	95% CI	*p*-Value
Age (yrs)	1.017	1.008–1.027	0.000	1.015	1.006–1.024	0.002
Sex						
Male	1					
Female	0.851	0.687–1.054	0.140			
Side						
Left	1					
Right	1.091	0.889–1.339	0.404			
Tumor Site						
Precentral	1					
Retrocentral	1.045	0.811–1.347	0.733			
Temporal + Insular	1.031	0.810–1.312	0.806			
Radiological Features						
Ependymal involvement (*yes vs no*)	1.114	0.893–1.390	0.338			
Corpus Callosum involvement (*yes vs no*)	0.917	0.737–1.142	0.439			
Necrotic-cystic component (*yes vs no*)	0.974	0.781–1.215	0.816			
Midline shift (*yes vs no*)	0.979	0.797–1.202	0.838			
Preoperative Tumoral Volume computed on postcontrast T1-weighted images, cm^3^	1.003	0.999–1.008	0.170			
Preoperative Tumoral Volume computed on T2-weighted images, cm^3^	0.996	0.994–0.998	0.000	0.999	0.996–1.001	0.311
Preoperative ΔT1/T2 MRI Index	1.016	1.012–1.020	0.000	1.011	1.005–1.016	0.000
Residual tumor, cm^3^	1.083	1.067–1.100	0.000	0.977	0.943–1.013	0.208
EOR (continuous variable)	0.949	0.942–0.957	0.000	0.948	0.935–0.961	0.000
EOR (categorical variable)						
EOR = 100%	1					
99% ≤ EOR ≤ 90%	1.622	1.254-2.098	0.000			
89% ≤ EOR ≤ 80%	2.425	1.783-3.298	0.000			
EOR ≤ 79%	5.245	3.854-7.138	0.000			
Biological Features						
MGMT promoter methylation (*yes vs no*)	0.639	0.518-0.787	0.000	0.673	0.544-0.833	0.000
IDH 1/2 mutation (*yes vs no*)	0.706	0.488-1.023	0.066	0.894	0.612-1.305	0.561
Ki67	1.000	0.995-1.006	0.873			

Table showing the influence of different factors on the PFS rates as per univariate survival analysis and multivariate analysis on the entire GBM patients cohort. (*p*-value < 0.05 at Log-rank test). Boldfacing values represent statistical significant results (*p* < 0.05). CI = confidence interval; *p*-value = level of marginal significance; MRI = magnetic resonance image; preoperative ΔT1/T2 MRI Index = ratio between pre-operative tumoral volume on postcontrast T1-weighted and T2 weighted images; EOR = extent of resection; CWs = Carmustine Wafers; RT = radiotherapy; CT = chemoteraphy; MGMT = O^6^-methylguanine-DNA methyl-transferase; IDH = isocitrate dehydrogenase; OS = overall survival.

**Table 3:** One-year estimated overall survival and hazard ratios for each score with relative 95% confidence intervals. A score (GBM-associated prognostic score, GAPS) from 0 to 4 was then assigned to the 10 terminal nodes thus defined based on the relative hazard ratio (RHR).

Score	Variables	OS% (95% CI)	HR (95%CI)	*p*-Value
0	Preoperative ΔT1/T2 MRI Index < 0.72; EOR > 96%; Age < 53	92.24 (77.82–97.43)	1	-
1	Preoperative ΔT1/T2 MRI Index < 0.72; EOR > 96%; Age > 53	84.36 (77.39–89.33)	2.6 (1.4–5.0)	**0.003**
	Preoperative ΔT1/T2 MRI Index < 0.72; EOR: 81%–95%; Preop T2-w vol > 147 cm^3^			
2	Preoperative ΔT1/T2 MRI Index < 0.72; EOR: 81%–95%; Preop T2-w vol < 147cm^3^	43.85 (35.94–51.48)	9.6 (5.1–18.3)	**0.000**
	Preoperative ΔT1/T2 MRI Index > 0.72; EOR >91%			
	EOR: 56%–80%; Age < 59			
3	Preoperative ΔT1/T2 MRI Index > 0.72; EOR 81%–90%	11.58 (5.69–19.76)	28.1 (14.4–54.7)	**0.000**
	EOR: 56%–80%; age > 60			
4	EOR < 55%	5.26 (0.36–21.43)	85.4 (38.5–189.3)	**0.000**

**Table 4:** One-year estimated PFS according to GAPS score.

GAPS Score	1-Year Estimated PFS
**Score 0**	65.60%
**Score 1**	55.54%
**Score 2**	13.09%
**Score 3–4**	3.93%

## Appendix A

**Table A1:** Baseline characteristics of the study population.

Parameters	Value (N and %, Mean ± Standard Deviation (SD) or Median and Range)
No. of patients	465
Age (years)	63 (20–85)
Sex	
Female	176 (37.85%)
Male	289 (62.15%)
Side	
Left	228 (49.03%)
Right	237 (50.97%)
Tumor Site	
Precentral	182 (39.14%)
Postcentral	128 (27.53%)
Temporal + Insular	155 (33.33%)
Intra-operative protocol	
CEUS + / 5-ALA +	43 (9.25%)
CEUS - / 5-ALA +	35 (7.53%)
CEUS + / 5-ALA -	34 (7.31%)
CEUS - / 5-ALA -	353 (75.91%)
Radiological Features	
Ependymal involvement (*yes vs. no*)	143 vs. 322 (30.75% vs 69.25%)
Corpus Callosum involvement (*yes vs. no*)	155 vs. 310 (33.33% vs 66.67%)
Necrotic-cystic component (*yes vs. no*)	319 vs. 146 (68.60% vs 31.40%)
Midline shift (*yes vs no*)	222 vs. 243 (47.74% vs 52.26%)
Preoperative Tumoral Volume computed on postcontrast T1-weighted images, cm^3^	31 (0.682–136)
Preoperative Tumoral Volume computed on T2-weighted images, cm^3^	65 (3–497)
Preoperative ΔT1/T2 MRI Index	48.55 (1.13–100)
Residual tumor, cm^3^	0.959 (0–37.506)
EOR (continuous variable)	95 (38–100)
EOR (categorical variable)	
EOR = 100%	184 (39.57%)
99% ≤ EOR ≤ 90%	133 (28.6%)
89% ≤ EOR ≤ 80%	76 (16.34%)
EOR ≤ 79%	72 (15.48%)
Biological Features	
MGMT methylation (*yes vs no*)	290 vs. 175 (62.37% vs. 37.63%)
IDH 1/2 mutation (*yes vs no*)	38 vs. 427 (8.17% vs. 91.83%)
Ki-67	25 (2-95)
Two-gene model	
MGMT met and IDH 1/2 mut	27 (5.81%)
MGMT met and IDH 1/2 wt	263 (56.56%)
MGMT unmet and IDH 1/2 mut	10 (2.15%)
MGMT unmet and IDH 1/2 wt	165 (35.48%)
Postoperative Protocol	
Stupp protocol	345 (74.2%)
Stupp protocol + CWs	60 (12.9 %)
Stupp interrupted for side effects	60 (12.9 %)

Features of the study population are described using means ± standard deviation or median and range for continuous variables, number of cases with relative percentages reported in parentheses for categorical variables. EOR = extent of resection; MRI = magnetic resonance image; preoperative ΔT1/T2 MRI Index = ratio between pre-operative tumoral volume on postcontrast T1-weighted and T2 weighted images; CWs = Carmustine Wafers; MGMT =O^6^-methylguanine-DNA methyl-transferase; IDH = isocitrate dehydrogenase.

**Table A2:** Clinical and follow-up characteristics of the study population.

	Value (N and %, Mean ± SD or Median and Range)
Clinical presentation	
No deficits	41 (8.82%)
Not-specific symptoms (headache, nausea, vomiting, disorientation etc.)	165 (35.48%)
Motor deficits	89 (19.14%)
Sensory deficits	16 (3.44%)
Visual/speech deficits	66 (14.19%)
Seizures	88 (18.92%)
Post-operative course	
No deficits	263 (56.56%)
Not-specific symptoms (headache, nausea, vomiting, disorientation etc.)	64 (13.76%)
Motor deficits	80 (17.20%)
Sensory deficits	3 (0.65%)
Visual/speech deficits	52 (11.18%)
Seizures	3 (0.65%)
6-monts follow-up (in 394 pts alive)	
No deficits	245 (62.18%)
Not-specific symptoms (headache, nausea, vomiting, disorientation etc.)	94 (23.86%)
Motor deficits	35 (8.88%)
Sensory deficits	1 (0.25%)
Visual/speech deficits	17 (4.31%)
Seizures	2 (0.51%)
KPS	
Pre-operative	90 (50–100)
Immediate post-operative	90 (50–100)
6-monts follow-up (in 394 pts alive)	90 (50–100)
OS (alive vs dead)	158 vs. 307 (33.98% vs. 66.02%)
OS at 1-year follow-up	54.78%
OS at 2-year follow-up	22.28%
PFS (no recurrence vs recurrence)	96 vs. 369 (20.65% vs. 79.35%)
PFS at 1 year follow-up	33.05%
PFS at 2 year follow-up	13.82%

Characteristics of the study population are described using means ± s.d. (standard deviation) or median and range for continuous variables, number of cases with relative percentages reported in parentheses for categorical variables. KPS = Karnofsky Performance Status; OS = overall survival; PFS = progression-free survival.
